# Potential role of corneal epithelial cell-derived exosomes in corneal wound healing and neovascularization

**DOI:** 10.1038/srep40548

**Published:** 2017-02-06

**Authors:** Kyu-Yeon Han, Jennifer A. Tran, Jin-Hong Chang, Dimitri T. Azar, James D. Zieske

**Affiliations:** 1 Department of Ophthalmology and Visual Sciences, Illinois Eye and Ear Infirmary, College of Medicine, University of Illinois at Chicago, Chicago, IL, United States; 2 Schepens Eye Research Institute/MEE, Boston, Massachusetts, United States; 3 Ophthalmology, Harvard Medical School, Boston, Massachusetts, United States

## Abstract

Specific factors from the corneal epithelium underlying the stimulation of stromal fibrosis and myofibroblast formation in corneal wound healing have not been fully elucidated. Given that exosomes are known to transfer bioactive molecules among cells and play crucial roles in wound healing, angiogenesis, and cancer, we hypothesized that corneal epithelial cell-derived exosomes may gain access to the underlying stromal fibroblasts upon disruption of the epithelial basement membrane and that they induce signaling events essential for corneal wound healing. In the present study, exosome-like vesicles were observed between corneal epithelial cells and the stroma during wound healing after corneal epithelial debridement. These vesicles were also found in the stroma following anterior stromal keratectomy, in which surgical removal of the epithelium, basement membrane, and anterior stroma was performed. Exosomes secreted by mouse corneal epithelial cells were found to fuse to keratocytes *in vitro* and to induce myofibroblast transformation. In addition, epithelial cell-derived exosomes induced endothelial cell proliferation and *ex vivo* aortic ring sprouting. Our results indicate that epithelial cell-derived exosomes mediate communication between corneal epithelial cells and corneal keratocytes as well as vascular endothelial cells. These findings demonstrate that epithelial-derived exosomes may be involved in corneal wound healing and neovascularization, and thus, may serve as targets for potential therapeutic interventions.

Wound healing is a complex process that involves hemostasis^1^, inflammation^2^,^3^, cell proliferation^4^, and remodeling of the local tissue environment^5^. Coordinated efforts of different tissues and cell lineages are required for replacement/repair of missing cellular structures and tissue layers^6^. The corneal wound healing process has been shown to follow the common progression of general wound healing^7^. This process requires epithelial cell proliferation and migration, stromal cell death, keratocyte proliferation, myofibroblast generation, collagen deposition, and inflammatory cell infiltration. Corneal wound healing has been studied extensively in the context of incisional surgery, excimer and thermal laser surgeries, epithelial debridement, and keratolysis (e.g., iatrogenic injury and noninfectious ulcers). It is known that the interaction between the corneal epithelium and stroma is important for transformation of keratocytes into myofibroblasts in incisional wound healing^8^. This intercellular signaling between epithelial and stromal cells is mediated by cytokines, neuropeptides, growth factors, chemokines, and matrix metalloproteinases, and through such factors, corneal epithelial cells stimulate keratocyte proliferation in corneal wound areas^9^,^10^,^11^. Molecules such as TGF-β, PDGF, HGF, and KGF have been known as key players in the wound healing cascade. However, the interactions of epithelial cell-derived exosomes within the injured stroma have yet to be identified, and how corneal clarity is maintained after injury or surgery is poorly understood.

Exosomes, byproducts of endocytosis, are released by most cell types^12^ and range in diameter from 30–150 nm^13^,^14^,^15^. Cells shed microvesicle-containing surface membranes under both normal and pathological conditions. Exosomes also are secreted from cells by fusion with the plasma membrane via the endosomal vesicle pathway^16^. Exosomes contain bioactive molecules such as proteins, lipids, mRNAs, and microRNAs for the transfer of genetic information. Upon fusion of exosomes to target cells, these bioactive molecules are transferred, which leads to phenotypic changes in the target cells^17^. Therefore, exosomes represent good diagnostic and therapeutic candidates in the early stages of many diseases. Exosomes have been shown to play crucial roles in the pathological stages of angiogenesis^18^, immunosuppression^19^, and cancer. Relatively increased amounts of exosomes have been found in tumors in comparison with normal cells^20^ and are linked to the autocrine loop, extracellular matrix remodeling, and cancer progression^21^. Our previous research demonstrated that mouse corneal fibroblasts secrete exosomes^22^, but the activity of these exosomes remains unclear in the context of corneal wound healing and angiogenesis.

In this study, we characterized human and mouse corneal epithelial cell-derived exosomes and investigated the role of mouse-derived exosomes in intercellular communication between the epithelium and stroma during corneal wound healing. Our findings indicate that epithelial cell-derived exosomes may be important mediators of corneal wound healing and angiogenesis, and therefore, targets for novel prognostic and therapeutic strategies for treating corneal injury and preventing transplant rejection.

## Results

### Extracellular vesicles in wound area of rat and rabbit cornea

#### Rat epithelial debridement model

**Figure 1:**
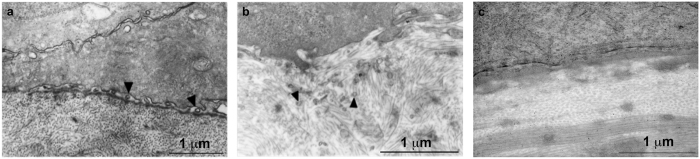
Transmission electron microscopy (TEM) images of wounded corneas with and without the basement membrane. (**a**) Rat cornea 18 hours after epithelial debridement. Small exosome-like vesicles (arrowheads) were present between corneal epithelial cells and the stroma. Magnification = 21,300x. (**b**) Rabbit cornea 48 hours after surgical removal of the epithelium, basement membrane, and anterior stroma. Small exosome-like vesicles were seen in the stroma (arrowheads). Magnification = 31,200x. (**c**) Normal rat cornea as control. Magnification = 30,000x.

Eighteen hours after rat corneas were subjected to epithelial debridement, exosome-like vesicles were detected in the wound area (Fig. 1a) between corneal epithelial cells and the stroma. Such vesicles were not observed in the stroma at 18 hours after removal of only the epithelial layer. We identified these vesicles as exosomes, rather than apoptotic bodies, based on their size (as opposed to apoptotic bodies that ranged from 1–5 μm in diameter). Electron microscopy of the non-wounded rat cornea did not reveal the presence of exosomes in the epithelial or stromal layers.

#### Rabbit anterior stromal keratectomy model

Exosome-like vesicles were found in the stroma 48 hours after the epithelium, basement membrane, and anterior stroma were surgically removed from the rabbit cornea (Fig. 1b). These findings are consistent with the hypothesis that the basement membrane serves as a barrier to prevent movement of epithelial-derived exosome-like vesicles to the stroma in healthy corneas.

### Characterization of exosomes isolated from human and mouse corneal epithelial cells and fibroblasts

**Figure 2:**
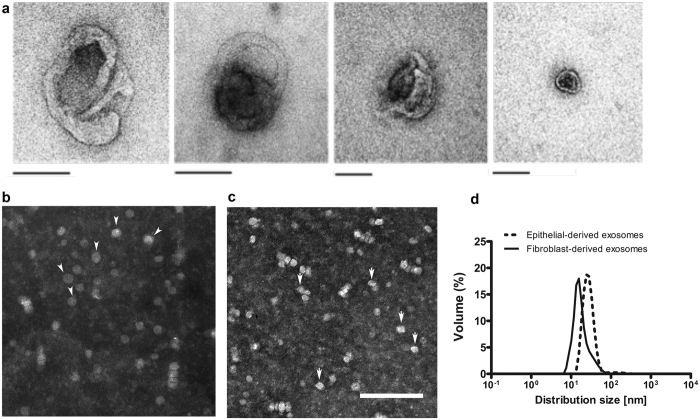
Characterization of human and mouse corneal epithelial cell-derived exosomes. Representative electron microscopy (EM) images of human corneal epithelial-derived exosomes (**a**) showing a range of exosomal morphologies. All scale bars indicate 100 nm. (**b**) EM image of mouse corneal epithelial cell-derived exosomes and (**c**) mouse corneal fibroblast cell-derived exosomes. (**d**) Size distribution of mouse corneal epithelial-derived exosomes. Dynamic light scattering (DLS) measurement of exosome size distribution.

Exosomes were purified from the conditioned media (CM) of human and mouse corneal epithelial cells as well as mouse corneal fibroblasts. After purification, the morphology of exosomes was evaluated using transmission electron microscopy (TEM). The human epithelial cell-derived exosomes demonstrated a range of morphologies (Fig. 2a), ranging from 30–150 nm in diameter. Similarly, the mouse corneal epithelial cells shed spheroid vesicles into the CM (Fig. 2b). Mouse corneal epithelial cell-derived exosomes appeared to be slightly larger than fibroblast-derived exosomes (Fig. 2c) The size distribution of mouse corneal epithelial-derived exosomes was further determined using dynamic light scattering (DLS) analysis (Fig. 2d). Z-average of mouse corneal epithelial cell-derived exosomes was 47.97 (d.nm) and mouse corneal fibroblast cell-derived was 34.22 (d.nm), and these exosomes had diameters ranging from 30–150 nm. The amounts of secreted exosomes isolated from the two mouse cell types were similar at 1 µg (just checking if mg is correct) per 1 × 10^7^ cells (data not shown).

**Figure 3:**
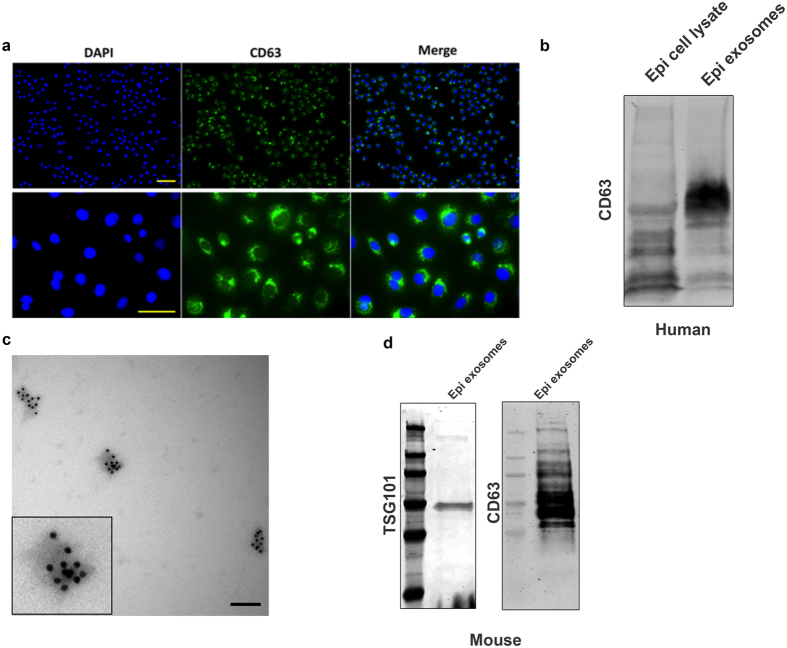
CD63 expression on human corneal epithelial cell cultures and exosomes. (**a**) Localization of CD63 expression on 1-day human corneal epithelial cell cultures. Cells are shown at low (top, 10x) and higher (bottom, 20x) magnification, and scale bars indicate 100 nm. (**b**) CD63 expression in western blot of human corneal epithelial cell lysate and exosomes collected from epithelial cell cultures and media. (**c**) Immuno-gold localization of CD63 on exosomes derived from human corneal epithelial cell cultures (Bar = 100 nm). (**d**) TSG101 and CD63 expression via western blotting of mouse corneal epithelial cell cultures.

In order to characterize the expression of exosomes in our samples, human corneal epithelial cells were cultured for 1 day and stained with DAPI and CD63, a membrane tetraspanin that is a common marker of exosomes. Virtually all DAPI-positive cells demonstrated CD63 expression, which appeared as punctate points surrounding the nuclei (Fig. 3a). Western blot results for human corneal epithelial cell lysate and exosomes isolated from 1-week cultures also showed positive staining for CD63 expression (Fig. 3b). Interestingly, the molecular weights of proteins identified by western blotting differed between the lysate and secreted exosome samples, suggesting that a processing of CD63 occurs before exosomes are secreted. Immunogold analysis revealed that CD63 was localized on isolated human corneal epithelial cell-derived exosomes (Fig. 3c).

We also assessed the expression of exosome makers CD63 and TSG101 on our mouse samples by western blotting and found that isolated exosomes from mouse corneal epithelial cells were enriched in these exosome marker proteins (Fig. 3d).

### Mouse corneal epithelial cell-derived exosomes fused to corneal fibroblasts

**Figure 4:**
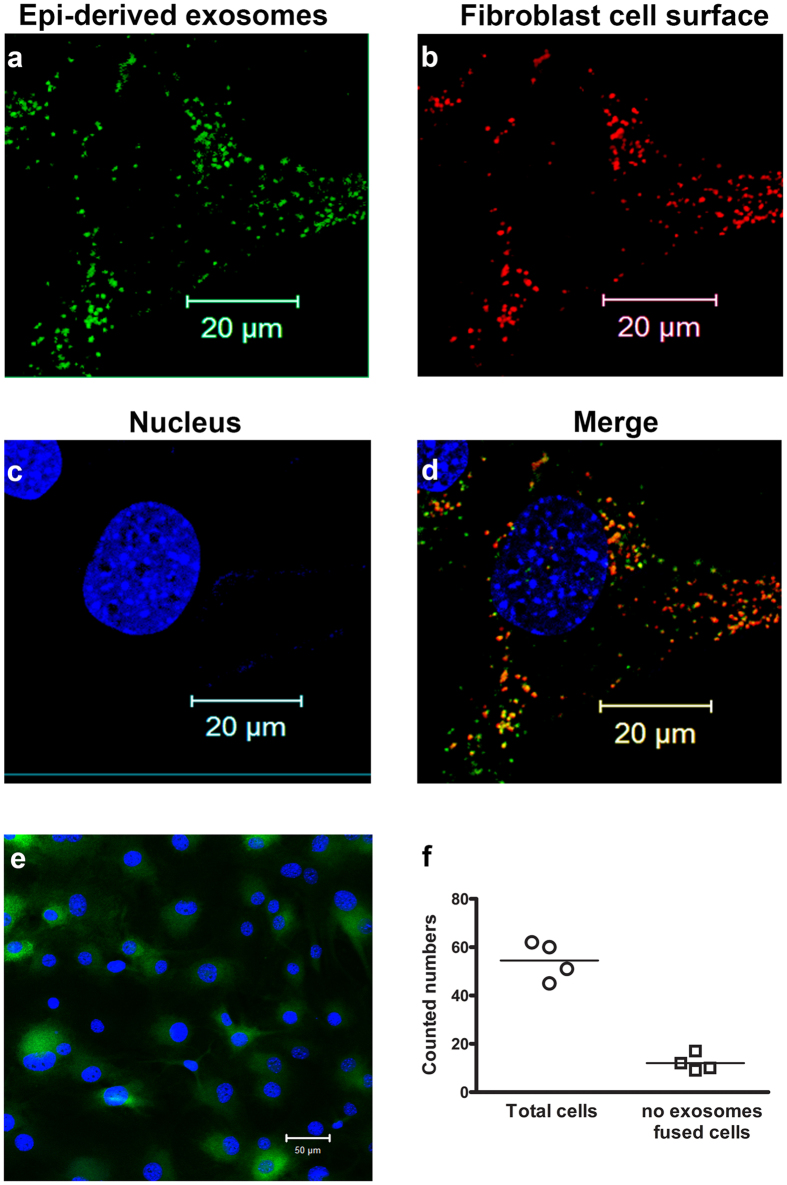
Confocal images of mouse corneal epithelial cell-derived exosomes fused to mouse corneal fibroblasts. (**a**) Isolated exosomes were labeled with a green fluorescent dye, and (**b**) the corneal fibroblast surface was stained with red fluorescent dye. (**c**) Nuclei were stained with DAPI. (**d**) Merged image showing all three stains. Scale bar indicates 20 μm. (**e**) Low magnification image, and (**f**) graphical representation of fusion ratio.

To investigate the exosome-based interaction between mouse corneal epithelial cells and fibroblasts, we labeled exosomes with green fluorescent dye (Fig. 4a) and the surface of fibroblasts with a red fluorescent dye (Fig. 4b). After incubation of cultured fibroblasts with labeled exosomes, green fluorescence was observed near the surface of fibroblasts. There appeared to be areas of colocalization between the labeled exosomes and the fibroblast surface, but most areas of green fluorescence did not overlap with red fluorescence (Fig. 4d). DAPI was used to stain nuclei, and no overlap of exosomes with nuclei was found (Fig. 4c and d). Low magnification (20x) images (Fig. 4e) were taken to calculate of percentage of exosomes fused to corneal keratocytes. All nuclei stained with DAPI were counted to determine the total cell number, and cells exhibiting green fluorescent were counted as exosomes fused to keratocytes. A fusion ratio of 77.13 ± 10.0% was obtained. Overall, our experiment demonstrated that epithelial cell-derived exosomes fused to fibroblasts.

### Effects of mouse corneal epithelial cell-derived exosomes on fibroblast proliferation

**Figure 5:**
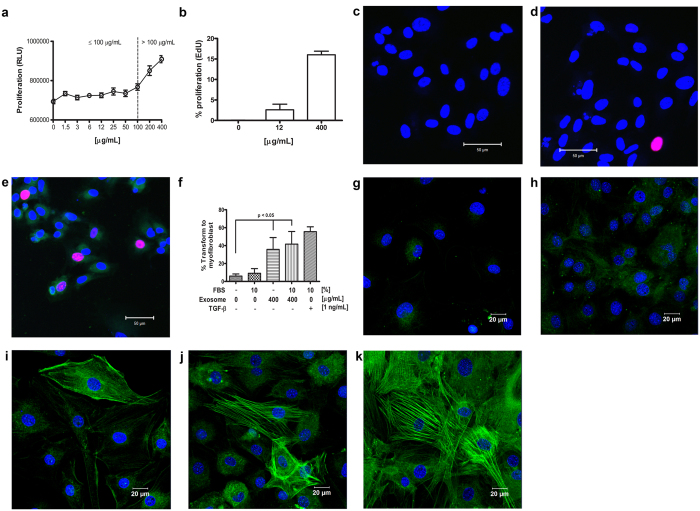
Effect of mouse corneal epithelial cell-derived exosomes on mouse corneal fibroblast proliferation and transformation into myofibroblasts. (**a**) Mouse corneal fibroblasts were incubated with various concentrations of mouse corneal epithelial cell-derived exosomes (1.5–400 μg/mL prepared at 2-fold serial dilution). The proliferation rate of mouse corneal fibroblasts was measured by incorporation of chemiluminescent BrdU. (**b**) Dividing keratocytes were identified and counted under confocal microscopy upon nuclear incorporation of EdU and staining with a fluorescent secondary antibody; (**c**) keratocytes without fluorescently labeled exosomes; (**d**) keratocytes exposed to 12 μg/mL fluorescently labeled exosomes, and (**e**) keratocytes exposed to 400 μg/mL fluorescently labeled exosomes. (**f**) Analysis of keratocyte transformation into myofibroblasts. Representative images of α-SMA immunostaining in cells cultured in serum-free medium (**g**), medium containing 10% FBS (**h**), medium containing 400 μg/mL exosomes (**i**), medium containing 400 μg/mL exosomes and 10% FBS (**j**), and medium containing 1 ng/mL TGF-β and 10% FBS (**k**). Scale bar indicates 20 μm. α-SMA was labeled with FITC, and nuclei were stained with DAPI for images in (**g**,**k**).

To investigate the biological functions of mouse corneal epithelial cell-derived exosomes in corneal wound healing, the effect of exosomes on mouse corneal fibroblast proliferation was evaluated. Activation of quiescent stroma keratocytes in the wound area is an important process in the repair of the injury site. Our results demonstrate that mouse corneal fibroblast proliferation was induced by mouse corneal epithelial cell-derived exosomes in a dose-response manner at concentrations greater than 100 μg/mL by BrdU analysis (Fig. 5a). To visually determine whether exosomes triggered keratocyte proliferation, EdU was used to distinguish dividing nuclei from quiescent nuclei. EdU incorporated in the nucleus was stained with alexa-647 secondary antibody, and all nuclei were stained with DAPI (Fig. 5b). Figure 5c shows a representative image of a keratocyte without fluorescently labeled-exosomes and with no detectable actively dividing nucleus. A low concentration of fluorescently labeled exosomes (12 μg/mL) started to induce keratocyte proliferation, and dividing nuclei stained with EdU were barely detected (Fig. 5d). We could observe a pink color upon merging the nuclear staining by EdU with keratocytes incubated with 400 μg/mL exosomes (Fig. 5e).

Activation of quiescent keratocytes involves the reorganization of cytoplasmic contractile proteins such as alpha-smooth muscle actin (α-SMA) within corneal wound healing. α-SMA expression was monitored on keratocytes cultured in serum-free medium, medium containing 10% FBS, and medium containing epithelial cell-derived exosomes. After 5 days, a series of phenotypic changes in stroma keratocytes was observed depending on the culture medium conditions. Epithelial cell-derived exosomes induced phenotypic changes of keratocyte transformation into myofibroblasts. Keratocytes cultured in serum-free medium showed that few cells expressed a basal level of α-SMA without reorganization of cytoplasmic contractile proteins (Fig. 5f and g, <5%). However, α-SMA expression was increased on keratocytes cultured in medium with 10% FBS for 5 days. However, no ultrastructural changes were observed in fibroblasts, with <10% of cells exhibiting smooth muscle-like characteristics (Fig. 5f and h). Interestingly, ultrastructural changes in α-SMA organization were observed (Fig. 5i) when medium containing epithelial cell-derived exosomes (400 μg/mL) was replaced by medium containing 10% FBS during keratocyte culture. In addition, keratocytes transformed into myofibroblasts when exosomes and medium with 10% FBS were used together (Fig. 5f and j, ±40%). Finally, 1 ng/mL transforming growth factor-beta (TGF-β) in medium with 10% FBS was used to induce transformation of keratocytes into myofibroblasts as a positive control (Fig. 5k). These results indicate that epithelial cell-derived exosomes contribute to the phenotypic changes that occur with stromal keratocyte transformation into myofibroblasts as well as the reorganization of cytoplasmic contractile proteins.

### Effect of mouse corneal epithelial cell- and fibroblast-derived exosomes on endothelial cell function

**Figure 6:**
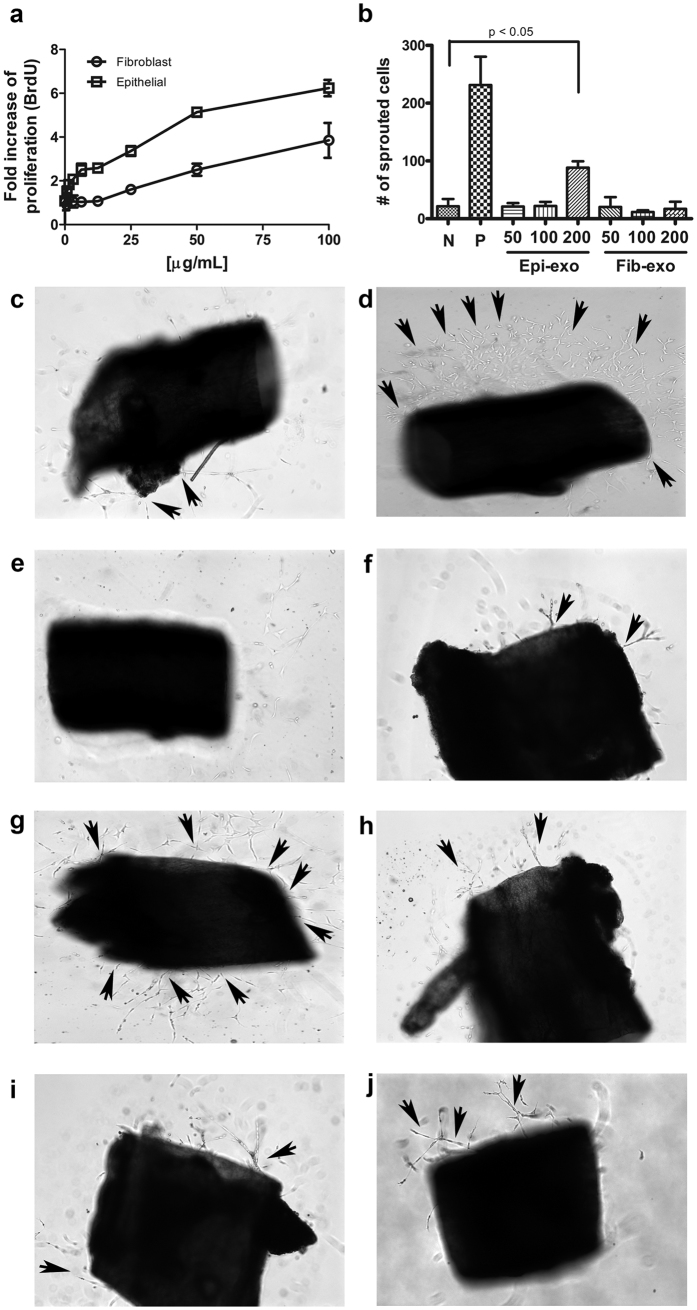
Effects of mouse corneal epithelial cell- and fibroblast-derived exosomes on angiogenesis. (**a**) Exosomes isolated from epithelial cells and fibroblasts stimulated endothelial cell proliferation in a concentration-dependent manner. (**b**) Endothelial microvessel sprouting from rings was detectable after stimulation with 200 μg/mL epithelial-derived exosomes. (**c–j**) Representative images of microvessel sprouting from aortic rings incubated in medium with 2.5% FBS (**c**), in EMB supplemented with growth factors (**d**), in medium containing 2.5% FBS and different concentrations of epithelial cell-derived exosomes ((**e**); 50 μg/mL, (**f**) 100 μg/mL, (**g**) 200 μg/mL), and in medium containing 2.5% FBS and different concentrations of fibroblast-derived exosomes ((**h**); 50 μg/mL, (**i**) 100 μg/mL, (**j**) 200 μg/mL).

We previously demonstrated that fibroblast-derived exosomes are taken up by vascular endothelial cells^22^. Here, we first evaluated the effect of mouse corneal epithelial- or fibroblast-derived exosomes on angiogenesis. Exosome-induced endothelial cell proliferation was investigated by BrdU incorporation. Human umbilical vein endothelial cells (HUVECs) were incubated with various concentrations of exosomes (1.5–100 μg/mL prepared at a 2-fold serial dilution). Interestingly, epithelial cell-derived exosomes induced HUVEC proliferation to a degree approximately 3-fold higher than that observed upon treatment with 100 μg/mL fibroblast-derived exosomes (Fig. 6a).

Next, *ex vivo* mouse aortic ring assays were used to monitor the effect of exosomes on endothelial cell growth (Fig. 6b). Mouse endothelial microvessel sprouting from aortic rings was observed after delivery of 200 μg/mL epithelial cell-derived exosomes (Fig. 6g). In contrast, fibroblast-derived exosomes induced significantly less microvessel sprouting than did epithelial cell-derived exosomes (Fig. 6j). EBM-2 medium supplemented with growth factors was used as a positive control (Fig. 6d), and medium alone was used as a negative control (Fig. 6c). Taken together, our results suggest that epithelial cell-derived exosomes may be more involved in endothelial cell proliferation than fibroblast-derived exosomes.

To further elucidate the biological activities of the epithelial cell-derived exosomes and given the differences in the effects of epithelial cell- and fibroblast-derived exosomes on angiogenesis, we performed exosome associated-protein profiling by proteomics analysis of the secreted exosomes from epithelial cells and fibroblasts using LC-MS/MS. We identified 139 proteins in fibroblast-derived exosomes and 44 proteins in epithelial cell-derived exosomes, with 33 proteins found in exosomes from both cell types. Proteins uniquely identified in epithelial cell-derived exosomes could be categorized broadly as extracellular binding, cell adhesion, and chemokine proteins (Table 1).

One of these was Col6a3 protein, which is known as an extracellular exosome component and might be involved in cell–cell communication. Other examples included C-X-C motif and C-C motif chemokines. Among the 33 overlapping proteins, syntenin and decorin are related to exosome biogenesis and secretion, respectively. In addition, three metalloproteinase-related proteins, MMP2, Tissue Inhibitor of MetalloProteinase 1 (TIMP1), and TIMP2, also were identified (Table 2).

**Figure 7:**
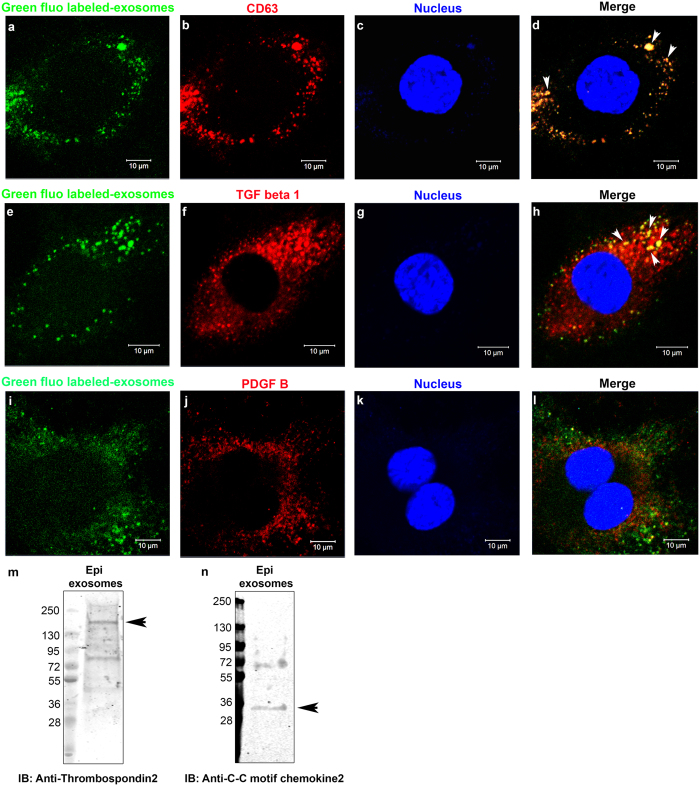
Characterization of mouse corneal epithelial cell-derived exosome-associated proteins. (**a**,**e**,**i**) Confocal images of fused fluorescently labeled-exosomes to keratocytes. (**b**) Confocal images of exosomal marker CD63 protein, (**f**) TGF-β, and (**j**) PDGF B stained with Alexa-647 conjugated secondary antibodies. (**c**,**g**,**k**) Confocal images of nuclei staining with DAPI. (**d**,**h**,**l**) Merged images for all colors showing colocalization of proteins (white arrows). Thrombospondin 2 (**m**) and C-C motif chemokine 2 (**n**) were uniquely identified in mouse corneal epithelial cell-derived exosomes by western blotting.

However, to identify the molecules involved in the induction of keratocyte transformation to myofibroblasts on mouse corneal epithelial cell-derived exosomes, key player proteins including TGF-β and PDGF were analyzed by immunohistochemistry of post-treated exosomes on keratocytes. Most of the post-treated fluorescently labeled exosomes carried a positive signal for the exosome marker protein CD63 (Fig. 7a–d). Interestingly, a TGF-β positive signal (Fig. 7f) was observed on post-treated fluorescently labeled exosomes, and then overlapping colors were indicated with white arrows (Fig. 7h). Another molecule known to regulate myofibroblast development, PDGF B was not localized on mouse corneal epithelial cell-derived exosomes (Fig. 7i–l). To verify the identified exosomal protein from proteomic analysis, two proteins of interest, including thrombospondin2 (Fig. 7m) and C-C motif chemokine 2 (Fig. 7n), were detected on isolated mouse corneal epithelial cell-derived exosomes by western blot analysis.

## Discussion

The cornea can be wounded by injury, inflammation, and incisional surgical approaches such as keratotomy and laser-*in-situ* keratomileusis (LASIK). Impaired healing of corneal wounds can lead to visual refractive errors^23^. The corneal epithelium is separated from the stroma by the epithelial basement membrane^24^. We hypothesized that in cases of corneal epithelial basement membrane injury, corneal epithelial cell-derived exosomes are likely to gain access to the underlying stromal fibroblasts and, thereby, may play an important role in corneal wound healing.

Corneal keratocytes are structurally flattened cells that account for 40% of the stroma volume in newborns, but only approximately 5% in the adult cornea^25^,^26^. Keratocytes interconnected through occasional desmosomal-like junctions have been studied by TEM and scanning electron microscopy^27^,^28^. In the normal cornea, keratocytes are quiescent, but during wound healing, keratocytes are activated and exhibit phenotypic changes, ultimately transforming into myofibroblasts^29^. Myofibroblasts are responsible for extracellular matrix deposition and organization as well as contractile protein production in wound healing. It is important to note that myofibroblasts can result from keratocyte corneal resident cell transformation or can be derived from bone marrow-derived cells. The process of keratocyte transformation is associated with cell proliferation, migration, and development of stress fibers. In the present study, intercellular communication through exosomes between corneal epithelial cells and keratocytes was investigated by observing fibroblast proliferation and keratocyte transformation into myofibroblasts upon exposure to exosomes isolated from epithelial cells. Our results demonstrated that epithelial cell-derived exosomes might be involved in the ultrastructural changes of keratocytes and likely modulate the activities of keratocytes as well as the bone marrow-derived progenitor cells in the cornea.

To change the phenotype of the target cell type, exosomes likely must bind and fuse to target cells. We observed fusion of exosomes to the stromal fibroblast cell surface using fluorescently labeled exosomes. Moreover, our results suggest that epithelial cell-derived exosomes may be involved in the transformation of stromal fibroblasts into myofibroblasts. Epithelial cell-derived exosomes alone in culture medium containing 10% FBS induced α-SMA reorganization and led to the generation of myofibroblasts.

We also showed that the molecular weight of the protein CD63, a common marker of exosomes, varies when comparing exosomes from cells and their conditioned media, suggesting that a processing event occurs as these nanovesicles are primed for release from the cell. This result provides a potential factor to consider when evaluating exosomes as biomarkers in diseases for which it is critical to identify those that are prematurely released from the cell.

The cornea is a highly specialized transparent tissue that remains avascular in order to transmit light. It is entirely devoid of blood vessels, and lymphatic vessels are essential in the normal cornea. The balance between angiogenic and anti-angiogenic factors in the corneal epithelium must be tightly regulated to maintain corneal avascularity^24^. In our study, epithelial cell-derived exosomes were found to have angiogenic activity both *in vitro* based on the induction of HUVEC proliferation and *ex vivo* in aortic sprouting assays.

Our proteomic results revealed that mouse corneal epithelial cell-derived exosomes carried proteins relevant to wound healing and neovascularization including thrombospondin-2, latent-transforming growth factor beta-binding protein 1, C-X-C motif chemokine 5, and C-C motif chemokine 2. C-C motif chemokine 2 is secreted by various cells and has been suggested to regulate cell growth and migration in both paracrine and autocrine manners^30^. The thrombospondin family of proteins is involved in early wound healing processes via activation of latent TGF-β. In particular, thrombospondin-2 may be expressed on avascular tissues such as the cornea and play a role in corneal wound healing^31^. Thrombospondin-2 is involved in various cellular events, including proliferation, migration, and angiogenesis^32^. Exosomal transport of thrombospondin-2 has been reported previously^33^. However, the function of thrombospondin-2–containing exosomes requires further study. TGF-β is known to trigger fibrosis and myofibroblast transformation. TGF-β was not found by proteomic analysis on mouse corneal epithelial cell-derived exosomes, but was nonetheless colocalized on some post-treated exosomes. Interestingly, a TGF-β binding protein, latent transforming growth factor binding protein-1 (LTBP-1), was present in epithelial cell derived-exosomes. LTBP forms a protein complex with TGF-β and TGF-β propeptide for the storage of latent TGF-β in the extracellular matrix. TGF-β could be transferred via exosomes in complex form with LTBP-1. One possible explanation is that the exosomes did not carry enough TGF-β to be identified on proteomics analysis, but contained multiple bioactive molecules, including TGF-β, involved in cell adhesion, metalloproteinases, and chemokines. It is possible that these factors may contribute to the observed activity of the epithelial-derived exosomes and their ability to fuse to target keratocytes, thereby disrupting their quiescence and inducing phenotypic changes to generate myofibroblasts.

## Methods

### Isolation of corneal stromal keratocytes and cell culture

Five C57 mice were sacrificed in accordance with NIH guidelines, and the corneas were isolated under a surgical microscope. The isolated corneas were incubated with 2% dispase I (Gibco, Grand Island, NY, USA) for 1 hour at 37 °C. The epithelial layers and basement membrane were removed with a scalpel under a surgical microscope. Then the remaining corneal tissue was chopped and incubated with 2% collagenase in Dulbecco’s Modified Eagle Medium (DMEM) for 2 hours at 37 °C. Dissociated cells were harvested by centrifugation at 1,500 rpm for 10 minutes, seeded on 35-mm^2^ dishes in DMEM containing 10% fetal bovine serum (FBS), and cultured until 90% confluence. Isolated primary keratocytes were used up to passage 6.

### Isolation of exosomes

Exosomes were isolated using Total Exosome Isolation Reagent (Invitrogen, Carlsbad, CA, USA). Briefly, mouse corneal fibroblasts and epithelial cells were cultured in DMEM supplemented with 10% FBS. Confluent cells were washed three times with phosphate-buffered saline (PBS), and then DMEM containing 1% exosome-depleted FBS was added. After 24 hours, the conditioned medium (CM) from the cultures was collected and centrifuged at 1,500 rpm for 10 minutes followed by another round of centrifugation at 3,000 rpm for 30 minutes to eliminate cell debris and macroparticles. The supernatant was concentrated by ultra-filtration with a 100-kDa molecular weight cut-off membrane (Millipore, Billerica, MA, USA). An equal volume of Total Exosome Isolation Reagent was then added, and the resulting solution was incubated overnight in a cold room. On the following day, samples were centrifuged at 10,000 × g for 1 hour and pellets were re-suspended in PBS followed by additional ultracentrifugation at 100,000 × g for 1 hour. After resuspension in PBS, the isolated exosomes were finally stored at −80 °C until use.

Exosomes from human corneal epithelial cells were isolated using the differential ultracentrifugation method as described by Thery *et al*.^34^. Human corneal epithelial cells, described previously, were obtained^35^ and cultured to 80% confluency in serum-free keratinocyte media (Sigma-Aldrich, St. Louis, MO, USA) with additional epithelial growth supplement. Cell culture CM was collected and centrifuged at 300 × g (1200 rpm, Thermo Scientific Centra CL3R) for 10 minutes to remove dead cells and debris. It was then filtered through 0.2-μm syringe filters to remove other debris and contaminants. The resulting sample was ultracentrifuged at 100,000 × g (40,000 rpm, Beckman Coulter-50.2 TI) for approximately 20 hours before being resuspended in PBS and stored at −80 °C until use.

### Electron microscopy (EM)

#### Exosomes

Exosome samples were prepared by a routine “Negative Staining” method, for subsequent transmission electron microscopy (TEM) examination. A 12–15 μL droplet of exosome solution was adsorbed to 300 mesh Formvar/carbon coated copper grids. The exosomes were then negatively stained using 2% aqueous phosphotungstic acid (PTA), blotted, and air dried, prior to examination with a JEOL JEM-1220 TEM, operating at an accelerating voltage of 80 kV^36^. Images were taken at 120,000x magnification.

#### Epithelial debridement and keratectomy models

Two rat corneas and three rabbit corneas were wounded via previously described debridement^37^ and keratectomy^38^ techniques, respectively. Corneas were fixed in Karnovsky’s for TEM post-enucleation and processed as published previously. An ultramicrotome (LKB; Bromma, Sweden) was used to cut the tissues at 60–90 Å thick. Samples were visualized with an electron microscope (Philips 410; Philips Electronics N.V., Eindhoven, The Netherlands).

#### Immunogold staining of isolated exosomes

Isolated exosomes were fixed for 1 hour at room temperature in 2% paraformaldehyde in 0.1 M sodium phosphate buffer. Five microliters of the exosomes were absorbed onto 200 mesh Formvar/carbon coated nickel grids (Electron Microscopy Sciences, Hatfield, PA, USA) for 20 minutes. After absorption, the grids were rinsed three times with PBS and then transferred to PBS/50 mM glycine (Sigma-Aldrich, St. Louis MO, USA) for washing. The grids were blocked in 5% bovine serum albumin (BSA) in 1× PBS for 10 minutes at room temperature. The grids were incubated at 4 °C (~18 hours overnight) in the primary antibody (1:200, purified rabbit anti-human CD63 H-193, Santa Cruz Biotechnology) diluted in 1% BSA/PBS or 1% BSA/PBS alone as a negative control. The grids were then rinsed in 0.1% BSA/PBS and washed six times with 0.5% BSA/PBS. Next, the grids were incubated in a ‘bridge’ antibody consisting of 1:200 goat anti-rabbit IgG (H + L), highly cross-adsorbed antibody (Sigma-Aldrich) diluted in 1% BSA/PBS for 30 minutes at room temperature. The grids were then rinsed in 0.5% BSA/PBS. Next, grids were incubated with nanogold conjugated Protein-G antibody at 1:20 dilution in 1%BSA/PBS for 30 minutes at room temperature and then rinsed 8 times with PBS. The grids were incubated in 1% glutaraldehyde in 0.1 M sodium phosphate buffer (Electron Microscopy Services, Hatfield, PA, USA) for 5 minutes. After rinsing in deionized water, the grids were contrasted in uranyl acetate (UA)-oxalate solution, pH 7 (UA, Electron Microscopy Services, Hatfield, PA) for 5 minutes. A drop of methyl cellulose-UA (1:10, viscosity: 25 cP, Sigma Aldrich) that was pre-cooled on ice for 10 minutes was transferred to the grids directly. The grids were blotted on filter paper and air-dried prior to imaging. The exosomes were observed using a FEI Tecnai G2 Spirit transmission electron microscope (FEI, Hillsboro, OR) at an accelerating voltage of 100 kV interfaced with an AMT XR41 digital CCD camera (Advanced Microscopy Techniques, Woburn, MA) for digital TIFF file image acquisition. Rabbit IgG (Vector Laboratories, Burlingame, CA) and CD63 lysate (Novus Biologicals, Littleton, CO, USA) were used as negative and positive controls, respectively.

### Analysis of exosome size distribution and concentration

To determine the size and polydispersity of the isolated exosomes, we used dynamic light scattering (Malvern zetasizer, Worcestershire, UK). Isolated exosomes were resuspended in PBS without Ca^2+^ or Mg^2+^. One hundred microliter suspensions of exosomes were added to a cuvette, and the air bubbles were carefully removed. Three scattering measurements for size and density were recorded.

### Western blotting

Isolated exosomes were boiled in sodium dodecyl sulfate (SDS) loading dye for 10 min and subjected to 4–20% SDS-polyacrylamide gel electrophoresis (PAGE). Separate proteins in bands on the gel were transferred to a nitrocellulose membrane, which was then blocked with 3–5% skim milk in Tris-buffered saline for 1 hour. TSG101 (Abcam, Cambridge, MA, USA) antibodies were diluted (1:1000) with blocking solution and then incubated for 1 hour. CD63 antibody, thrombospondin2, and C-C motif chemokine 2 (Abcam, Cambridge, MA, USA) was diluted at 1:200. Membranes were incubated with fluorescently tagged secondary antibody (Li-Cor, Lincoln, NB, USA), and protein bands were detected using the Li-Cor Odyssey system (Li-Cor).

### Exosome fusion assay

To track pithelial cell-derived exosomes, we stained exosomes with a green (PKH67, Sigma-Aldrich) or red fluorescent dye (PKH26, Sigma-Aldrich)^13^. Briefly, 200 μg of isolated exosomes were resuspended in 50 μL PBS and mixed with 1 × 10^−6^ M dye for 5 minutes. Unbound dye was quenched with an equal volume of exosome-depleted FBS. Labeled-exosomes were resuspended in 4 mL PBS (without Ca^2+^ and Mg^2+^) followed by ultracentrifugation at 100,000 × g for 1 hour. The pellets were re-suspended in 100 μL PBS and stored at 4 °C until further use. Corneal fibroblast cells were stained with red fluorescent dye (PKH26). Labeled fibroblasts were incubated with labeled exosomes for 4 hours and then washed with PBS to remove unbound exosomes. Mounting medium including DAPI (Vector Laboratories, Burlingame, CA, USA) was used to stain nuclei, and images were taken under a confocal microscope (Zeiss lsm 710, Jena, Germany).

### Immunofluorescent staining

Human corneal epithelial cells were seeded onto 4-well culture slides and incubated at 37 °C for 1 day. Cells were fixed in ice-cold 100% methanol for 10 minutes, washed three times with PBS, and incubated in 1% bovine serum albumin (BSA) solution for 1 hour at room temperature. CD63 primary antibody (Abcam, Cambridge, MA, USA) was applied overnight at 4 °C. Cells were then washed three times with PBS and incubated in fluorescein isothiocyanate (FITC)-conjugated secondary anti-mouse antibody (Jackson Immunology, West Grove, PA) for 1 hour at room temperature. Samples were washed with PBS and stained with DAPI mounting media (Vector Laboratories) before being visualized via fluorescence microscopy (Nikon).

Mouse corneal keratocyte cells were seeded onto 8-well culture slides. After 1 day, green fluorescent labeled exosomes from mouse corneal epithelial cell-derived were incubated for 6 hours. Cells were fixed in 4% paraformaldehyde/PBS for 15 minutes, washed with PBS, and incubated in 1% bovine serum albumin (BSA) solution for 1 hour at room temperature. TGF-β, and PDGF B primary antibodies (Abcam, Cambridge, MA, USA) was applied overnight at 4 °C and Alexa 647-conjugated antibody (Invitrogen, Carlsbad, CA, USA) was used as the secondary antibody. Nuclei were stained with DAPI included in the mounting media (Vector Laboratories).

### Proteomic analysis

Isolated exosomes sent for protein identification using Mass Spectrometry. Trypsin digestion was performed to cleave the proteins into peptides, and the peptide mixture was subjected to LC-MS/MS analysis using a Q-exactive mass spectrometer equipped with a proxeon nano-spray source, easy-LC II HPLC, and an auto-sampler (LC Packings). A full-scan MS (m/z 320–1800) experiment was performed, followed by MS/MS experiments on abundant ions detected in the full-MS scan. The collection of MS and tandem MS data were analyzed using the MASCOT data analysis program. Database was searched using MASCOT to identify the protein.

### Exosome-induced endothelial cell proliferation

Aliquots of 3 × 10^3^ human umbilical vein endothelial cells (HUVECs, ScienCell, Carlsbad, CA, USA) maintained in endothelial cell growth medium (EBM) including supplements (EBM-2, Lonza, Allendale, NJ, USA) were seeded in 96-well clear-bottom plates and incubated overnight. Isolated mouse corneal keratocytes were seeded in 96-well clear-bottom plates with DMEM containing 10% FBS. The following day, the cells were washed with PBS and then incubated with various concentrations (0.8–100 μg/mL) of exosomes from wild-type corneal fibroblasts or epithelial cells for 24 hours. Bromodeoxyuridine solution (BrdU, 10 M; Roche, Mannheim, Germany) was added for measurement of cell proliferation following the manufacturer’s instructions. Incorporated BrdU was detected with anti-BrdU-POD-conjugated antibody. The light emission of the proliferating cells was measured chemiluminescence using in a microplate luminometer (BioTek, Winooski, VT, USA). For imaging of dividing cells to verify exosome-induced proliferation, mouse corneal keratocyte cells were seeded onto 8-well culture slides. After 1 day, 10 μM EdU (Molecular Probes, Carlsbad, CA, USA) with green fluorescent labeled exosomes first isolated from mouse corneal epithelial cells were incubated for 6 hours. Incorporated EdU on the nuclei was detected according to the manufacturer’s instructions. Incorporated EdU in the nuclei was stained with Alexa Fluor 647 (Molecular Probes). Total nuclei were stained with DAPI contained in the mounting media (Vector Laboratories). Images were taken under a confocal microscope (Zeiss).

### *Ex vivo* aorta ring assay

Aortic ring assays were performed as described by Kojima *et al*.^39^. Briefly, aortas were obtained from five C57 wild-type mice. Fatty tissues were removed from aortic rings, and the rings were starved in M199 containing 1% FBS (exosome depleted) overnight. One-millimeter-long aorta specimens were cut and transferred to wells of 96-well plates coated with 100 μL growth factor-reduced Matrigel (BD Biosciences, San Jose, CA, USA). Each aortic ring was placed on its side on top of the Matrigel. Various concentrations of epithelial cell- or fibroblast-derived exosomes (0, 50, 100, or 200 μg/mL) in EBM containing 2.5% FBS were added, and the solutions were changed every other day. EBM including complement growth factor (Cambrex, Walkersville, MD, USA) was used as the positive control, and EBM containing 2.5% FBS without exosomes was used as the negative control. Six aorta rings per each group were photographed with a phase contrast microscope during the 10-day observation period, and tube formation was analyzed by counting the number of microvessels in images processed using ImageJ (National Institutes of Health, Bethesda, MD, USA).

## Additional Information

**How to cite this article:** Han, K.-Y. *et al*. Potential role of corneal epithelial cell-derived exosomes in corneal wound healing and neovascularization. *Sci. Rep.* **7**, 40548; doi: 10.1038/srep40548 (2017).

**Publisher's note:** Springer Nature remains neutral with regard to jurisdictional claims in published maps and institutional affiliations.

## Figures and Tables

**Table 1:** Proteins uniquely identified in mouse corneal epithelial cell-derived exosomes.

Molecular function	Biological process	Access number
Calcium, ion binding		
Protein C1s1	Complement activation	tr|E9Q6C2|
Thrombospondin-2	Cell adhesion	sp|Q03350|
Latent-transforming growth factor beta- binding protein 1	Aorta development	sp|Q8CG19|
Glypican-1	Fibroblast growth factor regulator, Exosome component	sp|Q9QZF2|
Endopeptidase inhibitor		
Col6a3*	Cell-surface interaction, Exosome component	tr|J3QQ16|
Antileukoproteinase	Immune response, Exosome component	sp|P97430|
Extracellular composition or binding		
Collagen alpha-1 (VI)	Cell differentiation, Exosome component	sp|Q04857|
Nidogen-1	Cell-matrix adhesion, Exosome component	sp|P10493|
Cell adhesion		
EMILIN-2	Cell adhesion	sp|Q8K482|
Chemokine		
C-X-C motif chemokine 5	Regulation of proliferation	sp|P50228|
C-C motif chemokine 2	Angiogenesis, Endocytic vesicle	sp|P10148|

**Table 2:** Proteins identified in both mouse corneal epithelial cell- and fibroblast-derived exosomes.

Molecular Function	Biological process	Access number
Extracellular matrix		
Collagen alpha-1 (I)	Collagen biosynthesis	sp|P11087|
Collagen alpha-1 (V)	“	sp|O88207|
Collagen alpha-2 (I)	“	sp|Q01149|
Collagen alpha-2 (V)	“	sp|Q3U962|
Collagen alpha-2 (IV)	“	sp|P08122|
Collagen alpha-2 (VI)	“	sp|Q02788|
Decorin	Regulator of angiogenesis	sp|P28654|
Biglycan	Vessel remodeling, Exosome component	sp|P28653|
Laminin subunit beta-1	Regulator of cell migration, Exosome component	sp|P02469|
Laminin subunit gamma-1	“	sp|P02468|
Peroxidasin homolog	Matrix organization, Exosome component	sp|Q3UQ28|
Peptidase activity		
Lysyl endopeptidase	Lysine catabolic process	sp|Q9HWK6|
Procollagen C-endopeptidase enhancer 1	Proteolysis, Exosome component	sp|Q61398|
Trypsin	Protease	sp|P00761|
Complement C1r-A subcomponent	Immune response, Exosome component	sp|Q8CG16|
Serine protease HTRA1	Regulator of cell growth, Exosome component	sp|Q9R118|
Pentraxin-related protein PTX3	Immune response	sp|P48759|
Inter-alpha-trypsin inhibitor heavy chain H2	Metabolic process, Exosome component	sp|Q61703|
Pigment epithelium-derived factor	Regulator of angiogenesis, Exosome component	sp|P97298|
Complement C3	Regulator of angiogenesis, Exosome component	sp|P01027|
Metalloproteinase related		
72 kDa type IV collagenase	Regulator of angiogenesis	sp|P33434|
Metalloproteinase inhibitor 2	Regulator of cell proliferation, Exosome component	tr|Q6PI17|
Metalloproteinase inhibitor 1	Regulator of cell proliferation, Exosome component	sp|P12032|
Cell-surface interaction		
Basement membrane-specific heparan sulfate proteoglycan core protein	Degradation extracellular matrix	tr|B1B0C7|
ADP binding, ATPase activity		
Transitional endoplasmic reticulum ATPase	Vesicle mediated transport, Exosome component	sp|Q01853|
Epidermal growth factor-activated receptor activity		
EGF-containing fibulin-like extracellular matrix protein 1	Regulator of cell proliferation, Exosome component	sp|Q8BPB5|
Calcium, ion binding		
Fibulin-1	Matrix organization, Exosome component	sp|Q08879|
Fibulin-2	Cell adhesion, Exosome component	sp|P37889|
Nidogen-2	Cell adhesion, Exosome component	sp|O88322|
Thrombospondin 1	Angiogenesis, Exosome component	tr|Q80YQ1|
Transcriptional repressor activity		
Adipocyte enhancer-binding protein 1	Regulator of transcription, Exosome component	sp|Q640N1|
Developmental process		
Olfactomedin-like protein 3	Multicellular organism development, Vesicle component	sp|Q8BK62|
Exosome biogenesis		
Syntenin-1	Exosome secretion	sp|O08992|
